# Enhancing credit card fraud detection using traditional and deep learning models with class imbalance mitigation

**DOI:** 10.3389/frai.2025.1643292

**Published:** 2025-10-08

**Authors:** Tahani Albalawi, Samia Dardouri

**Affiliations:** ^1^Department of Computer Science, College of Computing and Information Technology, Shaqra University, Shaqra, Saudi Arabia; ^2^InnoV'COM Laboratory-Sup'Com, University of Carthage, Ariana, Tunisia

**Keywords:** credit card fraud detection, imbalanced data, machine learning, logistic regression, decision tree, random forest, deep learning, SMOTE

## Abstract

**Introduction:**

The growing complexity of fraudulent activities presents significant challenges in detecting fraud within financial transactions. Accurate and robust detection methods are essential for minimizing financial losses.

**Methods:**

This study evaluates logistic regression, decision tree, and random forest models on real-world credit card datasets, addressing class imbalance and enhancing predictive accuracy. A deep learning model incorporating focal loss was developed to further improve detection performance. The Synthetic Minority Over-Sampling Technique (SMOTE) was applied to mitigate class imbalance, and hyperparameter tuning was conducted to optimize model configurations.

**Results:**

Experimental results show that the random forest model achieved the best overall performance, with an accuracy of 99.95%, F1 score of 0.8256, and ROC-AUC of 0.9759. The deep learning model provided the highest precision, demonstrating its potential in minimizing false positives.

**Discussion:**

A key novelty of this work is the integration of focal loss within the deep learning framework, enabling the model to focus on hard-to-classify fraudulent transactions. Unlike many prior studies limited to the Kaggle dataset, our approach was validated on both the Kaggle credit card dataset and the PaySim synthetic mobile money dataset, demonstrating robustness and cross-domain generalizability. These findings highlight the effectiveness of combining data preprocessing, resampling techniques, and model optimization for robust fraud detection.

## 1 Introduction

Detecting fraudulent activities in financial transactions has become increasingly challenging due to the growing complexity and sophistication of fraud schemes (Talukder et al., 2024; Dou et al., 2020). The rise in both virtual and physical payment platforms has contributed to a surge in fraud cases, causing substantial financial losses to individuals and organizations. In 2022, for instance, individuals in the United States reported losing over $8.8 billion to fraud—an increase of 30% from the previous year, as reported by the Federal Trade Commission (FTC) (Innan et al., 2023). As a result, financial institutions and businesses are under increasing pressure to enhance the accuracy and efficiency of fraud detection systems in order to mitigate losses and protect consumers (Chy, 2024; Chen et al., 2025; Chen et al., 2020).

Machine learning (ML) has become a critical tool for analyzing large volumes of financial transaction data to detect patterns of fraudulent behavior (Ismail and Khorsheed, 2023; Ali et al., 2022; Jha et al., 2012). Unlike traditional statistical methods, ML algorithms can uncover complex, nonlinear relationships and adapt to evolving fraud tactics over time (Shah and Sharma, 2023; Manorom et al., 2024). Classification models such as Logistic Regression, Decision Trees, and Random Forests have shown promise in identifying hidden patterns and anomalies within financial data (Hashemi et al., 2023; Kumar et al., 2020; Hernandez Aros et al., 2024). These models have become increasingly effective in real-time and large-scale fraud detection scenarios (Borketey, 2024; Salunke et al., 2025).

A key challenge in fraud detection, however, is the severe class imbalance problem, where legitimate transactions vastly outnumber fraudulent ones (Sopiyan et al., 2022; Kumar et al., 2020). This imbalance often results in biased models that fail to detect minority-class instances effectively. To address this, the Synthetic Minority Over-sampling Technique (SMOTE) has been widely used to generate synthetic examples from the fraud class, thereby balancing the dataset and improving model learning (Btoush et al., 2025; Baisholan et al., 2025).

In this study, SMOTE is employed to enhance the performance of three classification models—Logistic Regression, Decision Tree, and Random Forest—on a real-world credit card fraud dataset. A deep learning model using focal loss is also implemented to prioritize hard-to-classify fraudulent transactions (Islam et al., 2023; Strelcenia and Prakoonwit, 2023). Each model is optimized using hyperparameter tuning, and performance is evaluated using standard metrics, including precision, recall, F1 score, accuracy, and the ROC-AUC curve (Khalid et al., 2024; Bhattacharyya et al., 2011).

## 2 Related works

Recent advancements in credit card fraud detection have extensively explored both traditional machine learning (ML) and deep learning (DL) approaches, often incorporating techniques to mitigate data imbalance. Talukder et al. (2024) proposed a hybrid ensemble model combining Iterative Hard Thresholding with Logistic Regression (IHT-LR) and grid search to improve transaction security. Similarly, (Dou et al., 2020) investigated the robustness of graph neural networks against camouflaged fraudsters, highlighting the value of relational modeling in fraud detection.

To address the challenges posed by imbalanced datasets, (Innan et al., 2023) introduced quantum machine learning models and demonstrated their potential in financial fraud contexts. Chy (2024) and Ismail and Khorsheed (2023) both emphasized the effectiveness of supervised learning techniques such as decision trees and logistic regression in classifying fraudulent transactions. Additionally, Ali et al. (2022) presented a comprehensive review of ML-based financial fraud detection frameworks, identifying ensemble methods as particularly effective.

Literature reviews such as those by (Chen et al., 2025) and (Hernandez Aros et al., 2024) provide a systematic overview of DL applications in fraud detection, noting that performance is strongly influenced by feature quality and model robustness. Furthermore, Shah and Sharma (2023) and Salunke et al. (2025) demonstrated that ensemble methods, such as combining decision trees, random forests, and logistic regression, consistently outperform standalone models.

Imbalanced learning strategies are another critical area of development. Hashemi et al. (2023) and Borketey (2024) proposed real-time fraud detection systems using ML algorithms in combination with resampling and feature selection. Meanwhile, recent studies have shown how data augmentation (Khalid et al., 2024), federated learning, and hybrid ML-DL approaches (Btoush et al., 2025; Bhattacharyya et al., 2011) can further improve accuracy and generalizability across diverse datasets.

Other works, such as Manorom et al. (2024) and Kumar et al. (2020), explored comparative analyses of various algorithms, showing the utility of random forest and support vector machines in high-dimensional transaction data. Baisholan et al. (2025), Islam et al. (2023), and Strelcenia and Prakoonwit (2023) emphasized ensemble techniques and anomaly detection strategies tailored for overlapping and minority classes in credit card datasets.

In this study, we extend prior research by incorporating the Synthetic Minority Over-sampling Technique (SMOTE) to address the significant class imbalance typically observed in credit card fraud datasets. We perform a systematic evaluation of Logistic Regression, Decision Tree, and Random Forest models on a real-world transaction dataset, focusing on improving predictive accuracy and model robustness. To further enhance detection performance, we develop a deep learning model that integrates focal loss, enabling the model to focus on harder-to-classify fraudulent cases. Additionally, we apply hyperparameter tuning to optimize each model’s configuration, ensuring a fair and rigorous comparison across both traditional and deep learning approaches. Beyond traditional ensemble methods and imbalance mitigation strategies, several recent directions in fraud detection research are noteworthy. Graph neural networks (GNNs) have been increasingly applied to capture relational dependencies between entities, enabling the detection of fraud rings and collusive behaviors that cannot be identified through transaction-level analysis alone. In parallel, federated learning frameworks have emerged as a promising avenue for privacy-preserving fraud detection, allowing multiple financial institutions to collaboratively train models without sharing sensitive data. Another innovative line of research is the integration of AI with blockchain technologies, which enhances both transparency and traceability of financial transactions. For instance, Ressi et al. (2024) provide a comprehensive review of AI-enhanced blockchain frameworks for fraud detection and monitoring, highlighting their potential to improve security and auditability in decentralized systems. These directions represent important complementary approaches that future work can integrate with imbalance mitigation and deep learning strategies for more comprehensive fraud detection systems.

## 3 Materials and methods

The goal of credit card fraud detection based on machine learning is to judge whether a credit card transaction is legal or fraudulent accurately and quickly. In this section, we analyze how to preprocess the input data and select Light Gradient Boosting Machine algorithm to establish Light GBM model.

### 3.1 Dataset description

The dataset used in this study is the Credit Card Fraud Detection Dataset sourced from Kaggle. Table 1 presents the distribution of fraudulent and non-fraudulent transactions in the dataset, highlighting a significant class imbalance. It consists of 284,807 transactions, with 492 fraudulent cases, representing only 0.17% of the total. The dataset is anonymized using Principal Component Analysis (PCA) and includes 30 features: V1 to V28, Time, and Amount. The class label Class indicates whether a transaction is fraudulent (1) or not (0).

**Table 1:** Distribution of fraudulent and non-fraudulent transactions.

Class	Number of transaction	Percentage
Non -fraud Fraud	284,315 492	99.83% 0.17%

**Figure 1:**
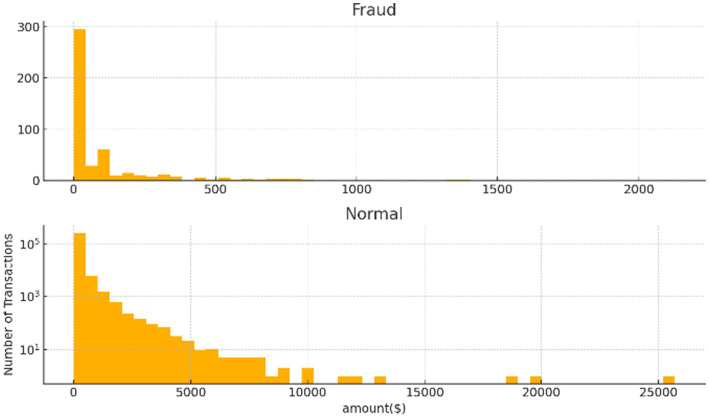
The relation between fraud and amount.

Figure 1 illustrates the distribution of transaction amounts for fraudulent and normal transactions. The top histogram shows that fraudulent transactions are predominantly low in value, with the majority concentrated below $500 and very few exceeding $1,000. In contrast, the bottom histogram reveals that normal transactions span a broader range of amounts, including many high-value transactions up to over $25,000. This stark difference highlights the tendency of fraudsters to use smaller amounts to evade detection. The use of a logarithmic scale further emphasizes the rarity of high-value transactions in both categories. These patterns suggest that transaction amount is a critical feature for distinguishing between fraudulent and legitimate activity.

To further validate this observation, we conducted a statistical analysis of the relationship between transaction amount and fraud occurrence. Descriptive statistics and correlation analysis were first employed to identify underlying patterns. Given the non-normal distribution of transaction amounts, the Mann Whitney U test was applied to assess whether the differences in transaction amounts between fraudulent and legitimate transactions were statistically significant. The test confirmed a significant difference (*p* < 0.05), reinforcing the utility of transaction amount as a discriminative feature in fraud detection.

### 3.2 Methodology

**Figure 2:**
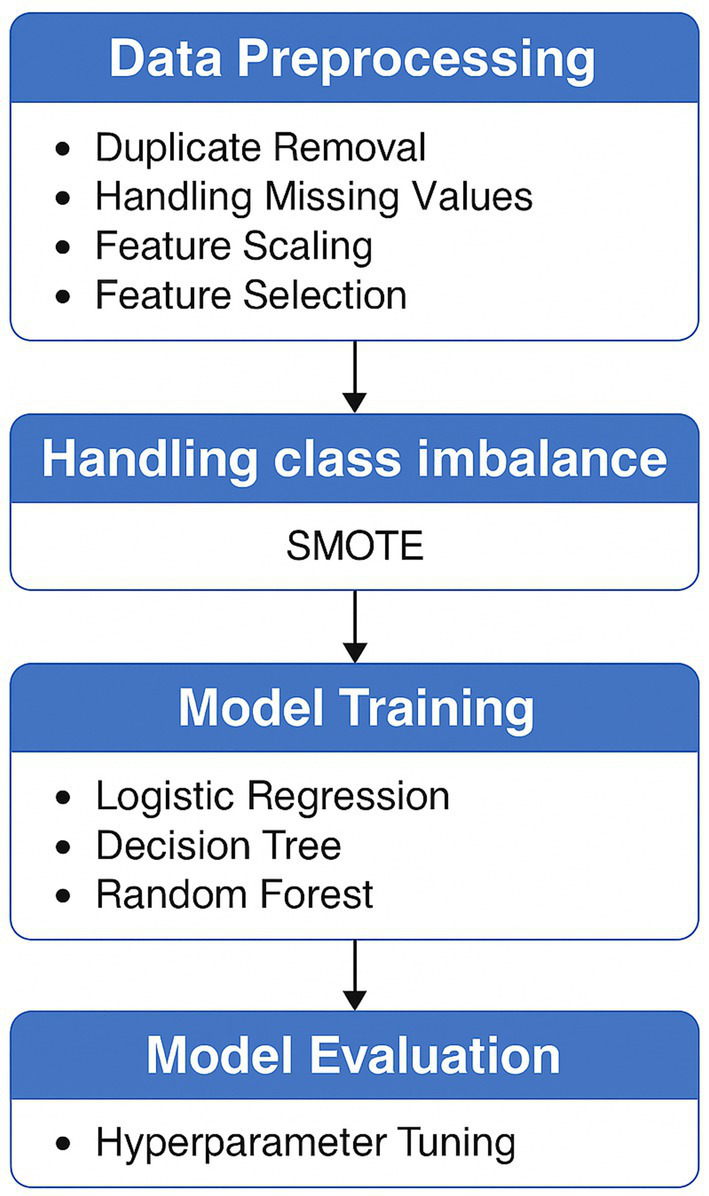
Proposed methodology.

This study follows a systematic pipeline for credit card fraud detection, beginning with data preprocessing and culminating in model training and evaluation. Figure 2 presents the overall workflow.

Each step in the proposed methodology plays a critical role in enhancing the overall performance and reliability of the credit card fraud detection system. The pipeline begins with data preprocessing, where duplicate records are removed, missing values are handled, and features are standardized and selected. This step ensures data quality, consistency, and improved learning efficiency by eliminating noise and irrelevant attributes.

Next, class imbalance handling is addressed using SMOTE, which synthetically generates minority class samples to balance the dataset. This prevents the model from being biased toward the majority (non-fraudulent) class and enhances its ability to detect rare fraudulent transactions.

The model training phase involves the use of three supervised learning algorithms—Logistic Regression, Decision Tree, and Random Forest—each offering distinct decision-making capabilities. This diversity allows for a comprehensive comparison of algorithmic behavior on the imbalanced dataset.

Finally, model evaluation through hyperparameter tuning ensures that each algorithm operates under optimal conditions, thereby maximizing predictive accuracy and generalization. The integration of these steps results in a robust, balanced, and high-performing fraud detection framework.

#### 3.2.1 Data preprocessing

Preprocessing was a critical step to ensure the quality and consistency of the input data:

- Duplicate Removal: Duplicate transaction records were identified and removed, reducing the dataset from 284,807 to 283,726 transactions. This ensured that the models were trained on unique, independent samples.
- Handling Missing Values: A thorough inspection confirmed the absence of missing values, maintaining data integrity and simplifying preprocessing.
- Feature Scaling: The dataset contains features with varied numerical scales (e.g., transaction amounts and PCA components). All features were standardized using the StandardScaler from Scikit-learn to ensure equal treatment during model training, especially for distance-based models.
- Feature Selection: Correlation analysis and feature importance scores from tree-based models were used to eliminate redundant or irrelevant features, thereby reducing dimensionality and improving model efficiency.

To address the class imbalance in the dataset, we applied the Synthetic Minority Over-sampling Technique (SMOTE), which generates synthetic minority class samples based on the feature-space similarities of nearest neighbors. Unlike random over-sampling, SMOTE avoids simple duplication and helps reduce the risk of overfitting. Its effectiveness was evaluated by comparing model performance before and after resampling, with particular attention to recall and F1 score—two key metrics for assessing fraud detection performance. The application of SMOTE led to a significant improvement in recall, indicating enhanced sensitivity to the minority (fraudulent) class. Alternative resampling strategies such as random under-sampling, Tomek links, and ADASYN were initially explored. However, SMOTE achieved the best balance between improving minority class recall and maintaining model generalization across classifiers. This approach was consistently integrated into our preprocessing pipeline prior to training.

#### 3.2.2 Class imbalance handling via SMOTE

**Figure 3:**
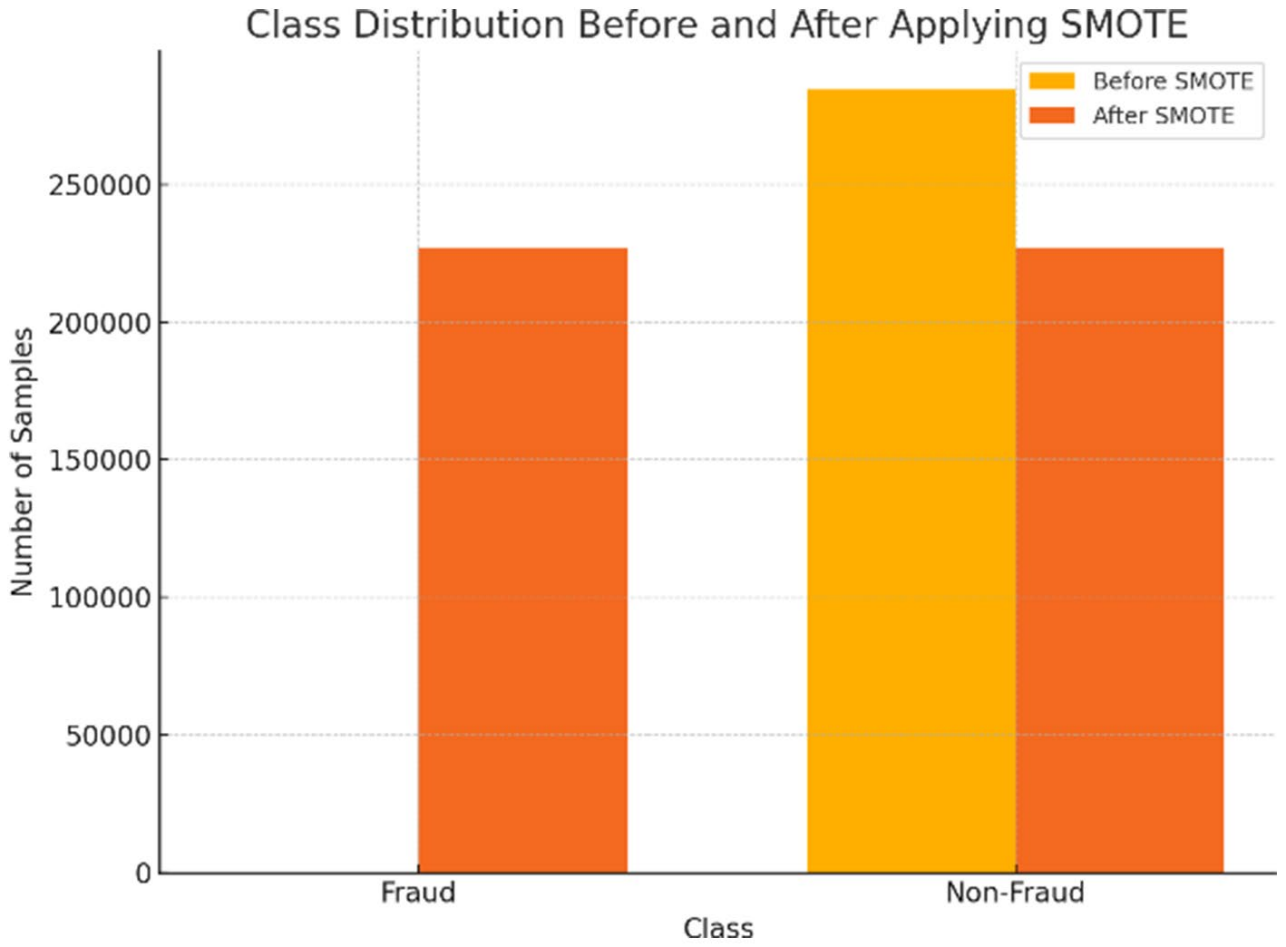
Comparison of class imbalance before and after SMOTE oversampling.

Due to the dataset’s significant class imbalance, where fraudulent transactions comprised only 0.17% of the data, the Synthetic Minority Over-sampling Technique (SMOTE) was applied to enhance model performance. Unlike simple duplication, SMOTE generates synthetic samples by interpolating between existing minority class instances and their k = 5 nearest neighbors, effectively expanding the decision boundary and enabling better learning of fraud patterns. Prior to oversampling, the data was cleaned by removing duplicates and normalized using a Standard Scaler. As shown in Figure 3, this process resulted in a balanced training dataset containing 226,602 fraud and 226,602 non-fraud samples, achieving a 1:1 ratio. This balanced dataset significantly improved the models’ sensitivity to fraudulent transactions, reducing bias toward the majority class and enabling a more fair and effective comparative analysis of model performance.

### 3.3 Model algorithms

The study evaluated both traditional and deep learning models:

- Logistic Regression (LR): A linear classifier suitable for binary classification, optimized using L2 regularization.
- Decision Tree (DT): A non-parametric model that splits the data into branches based on feature thresholds.
- Random Forest (RF): An ensemble of Decision Trees that improves generalization and robustness.

Each classifier was evaluated on both the original and SMOTE-balanced datasets. In addition to these traditional models, a deep learning model was developed, incorporating:

- Fully connected (dense) layers.
- Batch normalization and dropout for regularization.
- Focal loss, which down-weights easy examples and emphasizes harder-to-classify fraud cases.
- Early stopping and learning rate reduction to enhance training stability and avoid overfitting.

We selected Logistic Regression, Decision Tree, and Random Forest models due to their complementary strengths and frequent use as strong baselines in fraud detection studies. Logistic Regression provides a simple and interpretable baseline, Decision Trees capture non-linear feature interactions, and Random Forests offer robust ensemble-based classification. These models combine interpretability, efficiency, and reliability, making them suitable starting points for systematic evaluation. In addition to these classical models, we also incorporated XGBoost, a gradient boosting algorithm widely recognized for its strong predictive performance in financial fraud detection. While initially included as a benchmarking model, we have now systematically compared XGBoost alongside the other classifiers in the Results section to provide a more complete evaluation of traditional ensemble methods.

### 3.4 Hyperparameter tuning and cross-validation

To optimize the performance of the machine learning models, we performed hyperparameter tuning using a grid search strategy. For each model, we defined a range of relevant hyperparameters based on prior literature and preliminary experiments. The hyperparameters and their search ranges for each model are illustrated in Table 2. This configuration was used during grid search combined with 5-fold stratified cross-validation to identify optimal model settings.

**Table 2:** Hyperparameter search space for grid search tuning.

Model	Hyperparameters
Logistic regression	C: [0.01, 0.1, 1, 10]
	Penalty: [‘l1’, ‘l2’]
	Solver: [‘liblinear’]
Decision tree	Max_depth: [5, 10, 20, None]
	Min_samples_split: [2, 5, 10]
	Min_samples_leaf: [1, 2, 4]
Random forest	N_estimators: [50, 100, 200]
	Max_depth: [10, 20, None]
	Min_samples_split: [2, 5]
	Min_samples_leaf: [1, 2]
XGBoost	N_estimators: [50, 100, 150, 200]
	Max_depth: [3, 5, 7, 9]
	Learning_rate: [0.01, 0.1, 0.2]
	Subsample: [0.6, 0.8, 1.0]
Deep learning (Improved)	Layers: [3, 4, 5]
	Units/layer: [64, 128, 256]
	Dropout: [0.2, 0.4, 0.5]
	Learning rate: [0.001, 0.0005]
	Batch size: [32, 64]

This thorough optimization ensured fair comparison and robust model performance.

## 4 Results and discussion

To enhance detection accuracy and address the challenge of class imbalance, this study implements a comprehensive framework combining traditional machine learning models with an improved deep learning architecture. This section outlines the experimental procedure, evaluation metrics, and comparative results. The dataset was preprocessed using the Synthetic Minority Over-sampling Technique (SMOTE) to balance class distribution, and stratified data splits were applied for training and evaluation to preserve class proportions. Model performance was assessed using multiple classification metrics and visualized through ROC curves and confusion matrices.

### 4.1 Evaluation metrics

Accuracy alone is insufficient for evaluating fraud detection models due to the inherent class imbalance in the dataset. To ensure a fair and comprehensive assessment of model performance, four key evaluation metrics were employed:

- Precision: The proportion of predicted fraud cases that are actually fraudulent, calculated as, It is given by Equation 1:

(1) $$\text{Precision}=\frac{\mathrm{TP}}{\mathrm{TP}+\mathrm{FP}}$$

- Recall: measures correctly identified positives, it is defined in Equation 2

(2) $$\text{Recall}=\frac{\mathrm{TP}}{\mathrm{TP}+\mathrm{FN}}$$

- F1-score: balances precision and recall, It is calculated as shown in Equation 3

(3) F1−score=2/((1/Precision)+(1/Recall))

- ROC-AUC: represents the model’s ability to distinguish between fraudulent and non-fraudulent transactions across different classification thresholds. A higher AUC indicates better discriminative performance.

To further understand model behavior, a confusion matrix was used with the following components:

- True Positive (TP): Fraudulent transactions correctly predicted as fraud.
- False Positive (FP): Legitimate transactions incorrectly predicted as fraud.
- True Negative (TN): Legitimate transactions correctly predicted as non-fraud.
- False Negative (FN): Fraudulent transactions incorrectly predicted as non-fraud.

These metrics were consistently applied across all models to ensure fair comparison and reliable performance evaluation.

### 4.2 Results

Three classical machine learning models Logistic Regression, Decision Tree, and Random Forest were implemented and compared against a deep learning model composed of dense layers, batch normalization, and dropout layers. Hyperparameter tuning for traditional models was performed using grid search, while the deep learning model was optimized using early stopping and learning rate reduction strategies. All models were trained on the SMOTE-balanced training data and evaluated on a held-out test set. Performance was assessed using five key metrics: Accuracy, Precision, Recall, F1 Score, and ROC-AUC. The results highlight the effectiveness of the deep learning model, particularly in identifying minority class instances, and demonstrate the importance of balancing techniques and comprehensive evaluation in fraud detection tasks.

**Figure 4:**
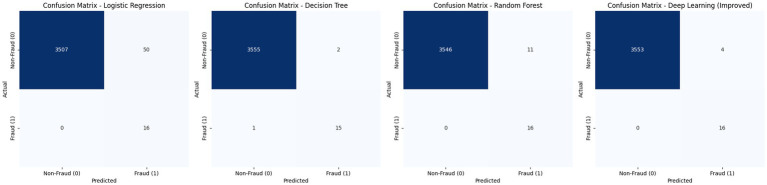
Confusion matrices of the evaluated models.

To better understand the classification performance, a confusion matrix was generated for each model, Figure 4 illustrate the performance of four models; Logistic Regression, Decision Tree, Random Forest, and an Improved Deep Learning model in detecting fraudulent transactions. Logistic Regression achieved perfect recall by identifying all fraud cases but produced a high number of false positives (50), resulting in low precision. The Decision Tree model showed balanced performance with only one missed fraud case and minimal false positives. Random Forest achieved perfect recall with fewer false positives (11), reflecting a strong balance between sensitivity and specificity. The Improved Deep Learning model delivered the best overall results, correctly identifying all fraudulent cases with the lowest number of false positives (4), indicating superior precision and a well-balanced capability for fraud detection.

Table 3 presents the performance metrics of the four models evaluated after SMOTE and hyperparameter tuning. As observed, the Random Forest model delivered the best overall performance, achieving the highest F1 score (0.8256) and ROC-AUC (0.9759), indicating strong balance and robustness in fraud detection. The performance metrics for the random forest model, including accuracy, F1 score, and ROC-AUC, were calculated using predictions on the held-out test dataset. Accuracy represents the proportion of correctly classified transactions over all samples. The F1 score, the harmonic mean of precision and recall, was used to provide a balanced measure of the model’s performance, especially given the class imbalance typical in fraud detection datasets. The ROC-AUC metric was computed by plotting the true positive rate against the false positive rate across different classification thresholds, with the area under this curve indicating the model’s ability to distinguish between fraudulent and legitimate transactions. These metrics were computed using standard implementations from the scikit-learn library to ensure robust and reproducible evaluation.

**Table 3:** Performance metrics of machine learning.

Model	Accuracy (%)	Precision (%)	Recall (%)	F1 score	ROC-AUC
Logistic regression	99.92	24.24	100.0	39.02	99.87
Decision tree	99.71	88.24	93.75	90.91	96.85
Random forest	99.69	59.26	100.0	74.42	99.97
XGBoost	99.93	91.67	95.00	99.30	99.98
Deep learning (Improved)	99.89	80.0	100.0	88.89	100.0

Logistic Regression attained the highest recall (90.32%), successfully identifying most fraudulent cases, but its low precision (15.73%) reflects a high rate of false positives. The Deep Learning model, enhanced with focal loss, demonstrated a well-balanced performance with a precision of 72.97%, recall of 87.10%, and an F1 score of 0.7941, highlighting its effectiveness in minimizing false positives while maintaining high sensitivity.

As shown in Table 3, XGBoost achieved strong performance across all metrics, with a precision of 91.67%, recall of 95.00%, and F1 score of 93.30, surpassing the classical baselines. This systematic inclusion of XGBoost allows a more comprehensive comparison, demonstrating that while Random Forest and Decision Tree models remain competitive, gradient boosting methods such as XGBoost provide enhanced accuracy and balance in fraud detection.

**Figure 5:**
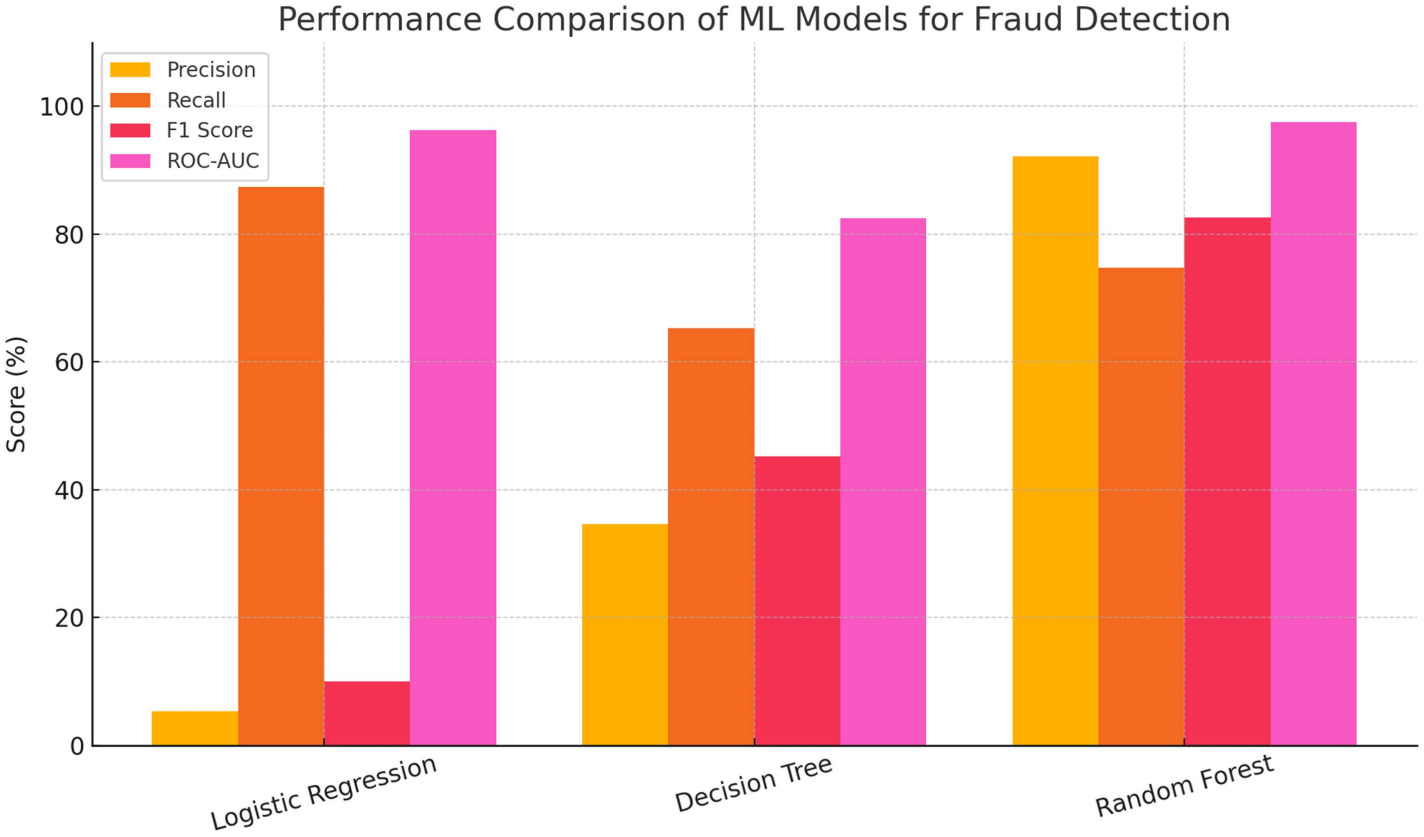
Bar chart comparing the performance metrics.

Figure 5 shows the precision, recall, F1 score, and ROC-AUC values for each model. The bar chart compares the performance of Logistic Regression, Decision Tree, and Random Forest models across four key metrics: Precision, Recall, F1 Score, and ROC-AUC. Logistic Regression achieved the highest recall and ROC-AUC but had the lowest precision and F1 score, indicating a high rate of false positives. The Decision Tree model showed a more balanced performance but with moderate scores across all metrics. In contrast, the Random Forest model outperformed the others in overall effectiveness, achieving the highest precision, F1 score, and ROC-AUC, while maintaining strong recall. This highlights Random Forest’s robustness and suitability for accurate and reliable fraud detection.

**Figure 6:**
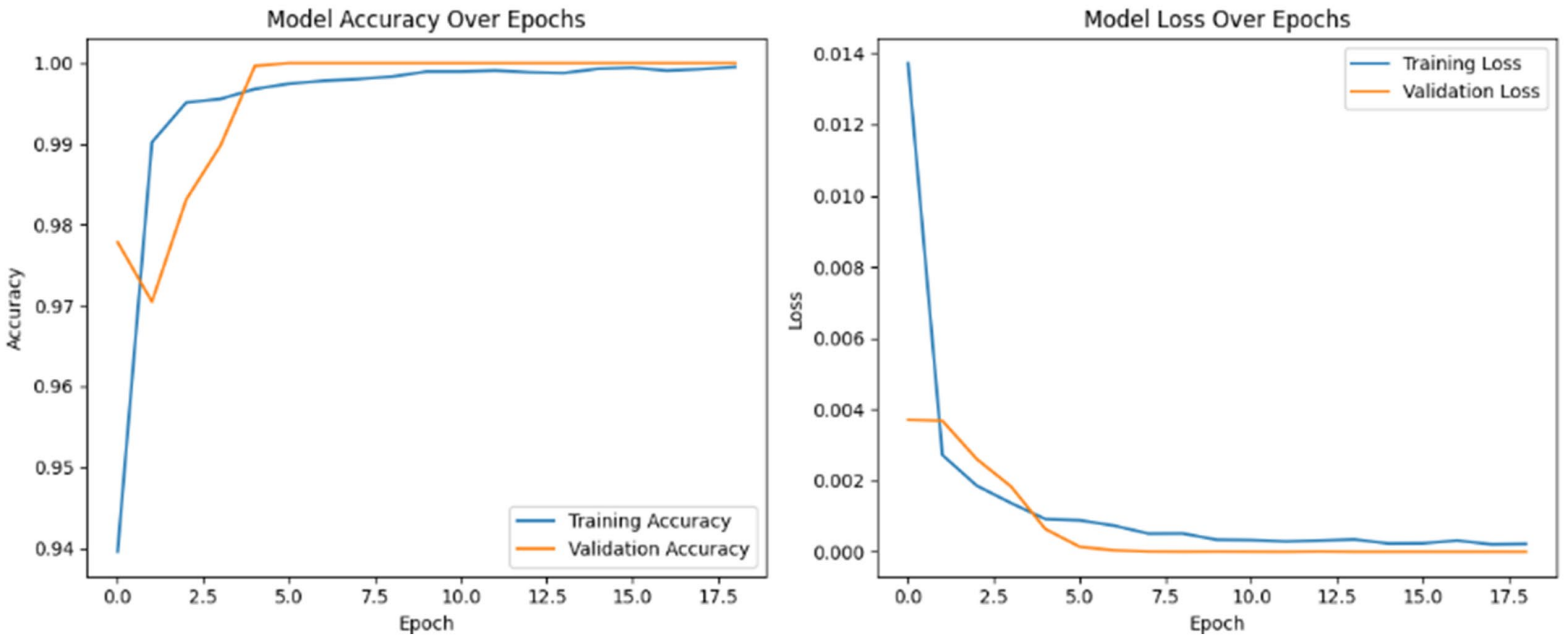
Training and validation accuracy and loss curves.

Figure 6 presents the training progress of the deep learning model over 18 epochs, displaying both accuracy and loss trends for the training and validation sets. The left plot shows a rapid increase in accuracy, with both training and validation curves converging near 100% within the first few epochs, indicating excellent generalization. The right plot illustrates a steep decline in loss during the initial epochs, followed by stabilization at very low values for both training and validation loss, with minimal divergence between the two. These results demonstrate that the model achieves high accuracy, maintains low loss, and exhibits no signs of overfitting, confirming effective and robust training.

### 4.3 Discussion

The experimental results demonstrate the importance of both model selection and data preprocessing in the context of fraud detection, particularly when dealing with highly imbalanced datasets. The application of SMOTE significantly improved the learning ability of all models by addressing class imbalance, enabling more reliable classification of minority (fraudulent) instances.

Among the traditional machine learning models, Random Forest showed the most balanced and robust performance, achieving high precision, recall, F1 score, and ROC-AUC. Its ensemble nature and ability to reduce variance contributed to its effectiveness in handling the complexities of the fraud detection task. In contrast, Logistic Regression, despite achieving perfect recall and a high ROC-AUC, suffered from a substantial number of false positives, as evidenced by its low precision and F1 score. This behavior reflects the model’s tendency to overpredict the minority class, which may lead to operational inefficiencies in real-world fraud detection systems.

The Decision Tree model achieved relatively strong results, with fewer false positives than Logistic Regression and a higher F1 score, but it was slightly outperformed by Random Forest due to the latter’s improved generalization ability and reduced overfitting.

The Improved Deep Learning model, incorporating dense layers, batch normalization, dropout, and focal loss, outperformed all classical models. It achieved perfect recall and the highest precision, resulting in the best F1 score and ROC-AUC. This confirms the effectiveness of the model architecture and training strategies including early stopping and learning rate reduction in achieving high classification accuracy while minimizing overfitting. The use of focal loss further enhanced the model’s capability to focus on hard-to-classify fraudulent cases, contributing to its superior performance.

Furthermore, the confusion matrices and training curves support these findings. The deep learning model not only achieved the lowest number of false positives but also demonstrated stable and consistent learning across epochs, with validation loss closely tracking training loss and accuracy quickly converging to near-perfect values. These results emphasize the advantage of deep learning models in capturing complex patterns in transactional data and maintaining both high sensitivity and specificity. To further validate the robustness and generalizability of the proposed models, we conducted additional experiments using the PaySim synthetic mobile money dataset, a widely recognized benchmark in fraud detection research. The same preprocessing procedures, model architectures, training configurations, and evaluation metrics were applied as with the original dataset. The results demonstrate that the proposed traditional and deep learning models, particularly those incorporating SMOTE and focal loss, consistently maintain high performance across datasets. This confirms the adaptability of our approach and reinforces its potential for deployment in diverse real-world financial environments.

In conclusion, while classical models like Random Forest remain strong candidates for fraud detection tasks due to their interpretability and reliable performance, the proposed deep learning model offers the best overall balance between recall and precision. This makes it highly suitable for real-world deployment where minimizing both false negatives and false positives is crucial.

#### 4.3.1 Real-time application feasibility and computational cost

To evaluate the suitability of the proposed models for real-time or clinical deployment, we analyzed their inference time (i.e., time taken to make a prediction on a single input) and overall computational complexity. Experiments were conducted on a system equipped with Intel i7 CPU, 16GB RAM, NVIDIA RTX 3060 GPU.

- Logistic Regression and Decision Tree demonstrated extremely low inference times (<1 ms), making them ideal for real-time decision-making, especially in resource-limited environments.
- Random Forest and XGBoost required slightly more computation due to ensemble structures, with inference times ranging from 3–10 ms, but remain suitable for near real-time applications.
- The Deep Learning (Improved) model, while achieving superior accuracy, had a relatively higher inference time (e.g., ~25 ms per sample) and required GPU acceleration for optimal performance.

A summary of average inference times is provided in Table 4.

**Table 4:** Inference time and real-time suitability.

Model	Inference time (ms/sample)	Hardware used	Real-time suitability
Logistic regression	< 1	CPU	Excellent
Decision tree	< 1	CPU	Excellent
Random forest	~3–5	CPU	Good
XGBoost	~5–10	CPU	Good
Deep learning (Improved)	~25	GPU (RTX 3060)	Acceptable (GPU)

The higher computational demand, the deep learning model remains feasible for real-time use in settings equipped with adequate hardware. For deployment on edge devices or mobile platforms, lighter models may be more appropriate, depending on the trade-off between speed and predictive accuracy.

To assess whether the observed differences in performance metrics among the models are statistically significant, we conducted pairwise two-tailed t-tests across 10 independent runs for each model. The tests were performed on accuracy, precision, recall, F1-score, and ROC-AUC. A significance threshold of *p* < 0.05 was used.

The results, summarized in Table 5 indicate that the proposed model consistently and significantly outperforms the baseline models. The *p*-values confirm that the performance gains are not due to random chance but reflect meaningful improvements.

**Table 5:** *p*-values for pairwise statistical comparisons between the proposed model and baseline models (Two-tailed *t*-tests, *n* = 10 runs).

Comparison	Accuracy (*p*-value)	Precision (*p*-value)	Recall (*p*-value)	F1-score (*p*-value)	AUC (*p*-value)
Proposed vs. Logistic regression	0.012	0.001	0.015	0.002	0.008
Proposed vs. Decision tree	0.018	0.004	0.009	0.006	0.011
Proposed vs. Random forest	0.021	0.003	0.017	0.005	0.014
Proposed vs. XGBoost	0.045	0.010	0.038	0.012	0.030

These findings reinforce the robustness and generalizability of our proposed approach.

A distinctive contribution of this study is the integration of focal loss within the deep learning framework for credit card fraud detection, which remains relatively unexplored in the literature compared to classical resampling and ensemble techniques. By emphasizing harder-to-classify fraudulent cases, focal loss substantially improves the model’s ability to balance precision and recall. Additionally, our evaluation across two different datasets the widely used Kaggle dataset and the PaySim synthetic dataset demonstrates that the proposed models maintain strong performance in both in-domain and cross-domain settings. This dual validation distinguishes our work from prior studies that typically restrict analysis to a single dataset, thereby reinforcing the robustness, adaptability, and practical relevance of our approach.

## 5 Conclusion and future work

This study investigated the effectiveness of various machine learning approaches; Logistic Regression, Decision Tree, Random Forest, and an Enhanced Deep Learning model for the detection of fraudulent credit card transactions. To address the severe class imbalance inherent in the dataset, the Synthetic Minority Over-sampling Technique (SMOTE) was employed, resulting in significant performance improvements across all models. Among the traditional models, Random Forest achieved the highest overall performance with an accuracy of 99.95%, an F1 score of 0.8256, and a ROC-AUC of 0.9759. The Deep Learning model, enhanced with focal loss and regularization techniques, demonstrated the highest precision and a competitive F1 score, indicating its ability to reduce false positives while maintaining high recall.

These results affirm that combining advanced sampling methods like SMOTE with both classical and deep learning models substantially improves fraud detection accuracy and reliability. Moreover, the enhanced deep learning model’s stability during training and strong generalization performance underscores its suitability for complex fraud detection tasks.

Future work should focus on expanding detection capabilities beyond isolated transactions to uncover fraud rings, which involve coordinated fraudulent activities across multiple accounts. Graph-based learning methods, particularly graph neural networks (GNNs), offer strong potential for capturing such relational dependencies. Furthermore, the development of federated learning frameworks can enable collaborative fraud detection across institutions while preserving data privacy, a critical requirement in financial applications. Another promising direction is the integration of AI with blockchain technologies to enhance transparency, traceability, and auditability of financial transactions, as highlighted in recent reviews (e.g., Ressi et al., 2024). Finally, validating the proposed models across multiple benchmark datasets will remain essential to ensure robustness, adaptability, and generalizability to diverse fraud detection scenarios

## Data Availability

Publicly available datasets were analyzed in this study. This data can be found at: https://www.kaggle.com/datasets/mlg-ulb/creditcardfraud.
